# The α2δ-1-NMDA receptor complex and its potential as a therapeutic target for ischemic stroke

**DOI:** 10.3389/fneur.2023.1148697

**Published:** 2023-04-20

**Authors:** Tao Wu, Shao-Rui Chen, Hui-Lin Pan, Yi Luo

**Affiliations:** ^1^Key Laboratory of Laboratory Medicine, Ministry of Education of China, School of Laboratory Medicine and Life Sciences, Wenzhou Medical University, Wenzhou, Zhejiang, China; ^2^Center for Neuroscience and Pain Research, Department of Anesthesiology and Perioperative Medicine, University of Texas MD Anderson Cancer Center, Houston, TX, United States; ^3^Department of Laboratory Medicine, Zhongnan Hospital of Wuhan University, Wuhan, Hubei, China

**Keywords:** NMDA receptors, excitotoxicity, ischemic stroke, α2δ-1, gabapentin, pregabalin

## Abstract

*N*-methyl-_D_-aspartate receptors (NMDARs) play a critical role in excitotoxicity caused by ischemic stroke, but NMDAR antagonists have failed to be translated into clinical practice for treating stroke patients. Recent studies suggest that targeting the specific protein–protein interactions that regulate NMDARs may be an effective strategy to reduce excitotoxicity associated with brain ischemia. α2δ-1 (encoded by the *Cacna2d1* gene), previously known as a subunit of voltage-gated calcium channels, is a binding protein of gabapentinoids used clinically for treating chronic neuropathic pain and epilepsy. Recent studies indicate that α2δ-1 is an interacting protein of NMDARs and can promote synaptic trafficking and hyperactivity of NMDARs in neuropathic pain conditions. In this review, we highlight the newly identified roles of α2δ-1-mediated NMDAR activity in the gabapentinoid effects and NMDAR excitotoxicity during brain ischemia as well as targeting α2δ-1-bound NMDARs as a potential treatment for ischemic stroke.

## 1. Introduction

Stroke is the second most common cause of death and the leading cause of morbidity and disability worldwide (1). Ischemic stroke accounts for 85% of stroke patients and results from cerebral ischemia and ischemia-reperfusion injury (2). Brain ischemia causes a complex series of pathophysiological events, including oxidative stress, inflammation, apoptosis, ionic imbalance, and excitotoxicity, with glutamate-gated *N*-methyl-_D_-aspartate receptor (NMDAR)-mediated excitotoxicity being a key factor (3–5). NMDARs are fundamental to both the physiology and pathology of the mammalian central nervous system (CNS), with dual roles in neuronal survival and death (6, 7). Normal NMDAR activity is essential for many neurological functions, including neuronal plasticity, brain development, and memory (8). Nevertheless, NMDAR over-activation can cause calcium overload, activating the downstream death-signaling pathways, and ultimately leading to irreversible neuronal death, which is called “excitotoxicity” (9–11). Although a number of animal studies have indicated that NMDAR blockers have neuroprotective effects on ischemic brain injury, NMDAR antagonists have proven largely unsuccessful in clinical trials mainly due to inhibiting physiological functions of NMDARs and intolerable side effects (12, 13). Further elucidating the molecular mechanism leading to pathological NMDAR hyperactivity during brain ischemia is essential for developing effective treatments for ischemic stroke. α2δ-1 (encoded by *Cacna2d1*), commonly known as a subunit of voltage-gated calcium channels (VGCCs), is a newly identified interacting protein of NMDARs in neuropathic pain (14–16). In this review, we briefly discuss recent findings about α2δ-1-bound NMDARs and their roles in excitotoxicity in ischemic stroke and the potential of targeting α2δ-1-bound NMDARs for treating cerebral ischemia.

## 2. Dual roles of NMDARs in neuronal survival and death

As the main subtype of ionotropic glutamate receptors, NMDARs are heterotetramers formed mostly by two GluN1 subunits and two GluN2 subunits (mainly GluN2A and GluN2B), serving as an important component of the excitatory post-synaptic membrane (17). Some studies suggest that activation of synaptic NMDARs may promote neuronal survival, whereas stimulation of extrasynaptic NMDARs may mediate pro-death effects (11, 18–20). However, this remains a hypothesis, and it is uncertain how the survival and death-signaling proteins are segregated to the subcellular synaptic or extrasynaptic sites (11). In this regard, post-synaptic density-95 (PSD95) is involved in NMDAR-mediated excitotoxic injury in synaptic sites (21). Moreover, it has been hypothesized that GluN2A-containing NMDARs are involved in neuronal survival, whereas GluN2B-containing NMDARs cause neuronal apoptosis and excitotoxicity (22). Contrary to this assumption, GluN2A-containing NMDARs can mediate neuronal death, and GluN2B-containing NMDARs may promote neuronal survival under certain experimental conditions (23). In the adult brain, GluN2A- and GluN2B-containing NMDARs may be preferentially localized at the synaptic and extrasynaptic sites, respectively (8). It is unclear how these NMDARs are differentially involved in the activation of the downstream neuronal survival-signaling complex (NSC) and/or the neuronal death-signaling complex (NDC) (Supplementary Figure 1).

NSC and NDC may be closely associated with the NMDAR channels either through protein–protein interactions or through spatial compartmentalization to synaptic or extrasynaptic sites (13, 24). Targeting protein–protein interactions required for NMDAR-mediated death-signaling pathways may be a promising strategy for effectively treating stroke.

## 3. NMDAR protein–protein interactions in ischemic stroke

The cytoplasmic C-terminal domains (CTDs) of NMDARs are distinct and contain specific motifs for interactions with a variety of scaffolding proteins, enzymes, and synapse-associated signaling proteins (26, 27). Due to the unique role of CTDs in the downstream intracellular signaling and synaptic retention of NMDARs, the altered binding of proteins with NMDAR subunits has been identified to be closely related to specific downstream signaling pathways and aberrant NMDAR synaptic localization in several disease states, such as cerebral ischemia (28–31).

Protein–protein interactions involving the CTDs of NMDAR subunits in experimental models of cerebral ischemia have been reported (28, 30). The GluN2B/PSD95/neuronal nitric oxide synthase (nNOS) complex may play a key role in driving excitotoxic signals in ischemic stroke (21, 32). PSD95 can couple with the CTDs of GluN2B to trigger the pro-death-signaling pathway, and cerebral ischemia may induce the interaction of the downstream nNOS with post-synaptic PSD95 at excitatory synapses, produce a toxic level of NO, and lead to neuronal death (21). Disrupting nNOS-PSD95 interaction via overexpressing the N-terminal amino acid residues 1-133 of nNOS (nNOS-N_1 − 133_) or a small-molecular inhibitor of nNOS-PSD95 interaction, ZL006, showed potent neuroprotective activity (21). Furthermore, cell-permeable peptides interfering with the PSD95/GluN2B interaction, such as the NA-1, a peptide sequence of the GluN2B CTD (KLSSIESDV), seem to reduce ischemic stroke (33). Moreover, the activation of the GluN2B/CaMKII cascade may increase CaMKII-dependent phosphorylation of acid-sensing ion channels (ASICs) in hippocampal neurons, which can result in an increased intracellular Ca^2+^ and the subsequent acidotoxic neuronal death (34). In the oxygen-glucose deprivation (OGD) condition, CaMKII inhibition or knockdown can produce a neuroprotective effect (35). Furthermore, death-associated protein kinase 1 (DAPK1) can interact directly with the CTD of GluN2B, which may be a therapeutic target for ischemic stroke (36, 37). Cerebral ischemia may promote the formation of the GluN2B/DAPK1 complex, activate DAPK1-dependent phosphorylation of GluN2B, and enhance the NMDAR channel conductance, leading to neuronal death (36). In a mouse model of experimental stroke, administration of a cell membrane—permeable NR2B_CT_ peptide—can protect neurons against cerebral ischemic insults (36, 38).

Although GluN2B-containing NMDARs, especially the CTD and phosphorylation of GluN2B, have been suggested to play a role in inducing NMDAR-dependent neurotoxicity, the interaction between GluN2A and metabotropic glutamate receptor 1 (mGluR1) C-terminus seems to be also important for excitotoxicity in a rat model of ischemic stroke (30, 39). The disruption of GluN2A/mGluR1 interaction protected neurons against NMDAR-mediated excitotoxicity and reversed NMDAR-mediated regulation of ERK1/2 (39). Both GluN2A and GluN2B subunits may form a complex with transient receptor potential cation channel subfamily M member 4 (TRPM4) at the extrasynaptic site (40).

## 4. α2δ-1 as a novel NMDAR-interacting protein

### 4.1. α2δ-1 and VGCCs

The VGCCs are fundamental regulators of intracellular calcium homeostasis, which are composed of pore-forming α1, auxiliary β, and α2δ subunits (41, 42). α2δ subunits belong to glycosyl-phosphatidylinositol (GPI)-anchored protein family, which in addition to being the binding site of gabapentinoids α2δ-1 and α2δ-2), were also identified as pain genes in a forward genetic screen (α2δ-3) (43– 46). Among them, α2δ-1 is strongly expressed in many brain regions, including the cerebral cortex and hippocampus, and α2δ-1 is preferentially localized in excitatory neurons (47, 48). However, quantitative proteomic analysis indicates that α2δ-1 has a weak interaction with VGCCs in the brain tissue (49). In addition, α2δ-1 ablation has no effect on the expression pattern of the VGCC α1 subunit or VGCC currents in the brain (50, 51).

### 4.2. α2δ-1 as a binding target of gabapentinoids

Gabapentinoids (i.e., pregabalin, gabapentin, and mirogabalin) are widely used to treat neuropathic pain and epilepsy in clinic (52–54). α2δ-1 and α2δ-2 are the binding target of gabapentinoids (55, 56). Compared with α2δ-2, gabapentinoids have a much higher affinity for α2δ-1 (56). The binding of gabapentinoids to α2δ-1, but not α2δ-2, is mainly responsible for its efficacy in neuropathic pain and epilepsy (15, 45, 47). Furthermore, α2δ-2 seems to be preferentially expressed in inhibitory interneurons, which may be related to the CNS side effects of gabapentinoids. However, gabapentinoids have no evident effect on VGCC activity or VGCC-mediated neurotransmitter release at presynaptic terminals (15, 57–59). Thus, the exact mechanisms underlying gabapentinoid actions are not known until recently.

### 4.3. The α2δ-1-NMDAR complex and neuropathic pain

NMDAR hyperactivity at the spinal cord level plays a central role in the development of chronic pain after nerve injury. Recent studies reveal that α2δ-1 can directly interact with NMDARs, forming a heteromeric complex through its C-terminal domain (15). In contrast, α2δ-1 seems to interact with VGCCs and thrombospondins via the von Willebrand factor type A domain on the N terminus (60). The functional significance of the α2δ-1-NMDAR complex has been demonstrated in various disease conditions, including neuropathic pain caused by traumatic nerve injury, chemotherapy, small-fiber neuropathy, calcineurin inhibitors, genetic and stress-induced hypertension, opioid-induced hyperalgesia and analgesic tolerance, opioid addiction, and ischemic brain injury (16, 25, 61–71) (Supplementary Table 1). Mechanistically, α2δ-1 preferentially binds to phosphorylated NMDARs and promotes surface and synaptic trafficking of NMDARs, and also reduces the Mg^2+^ block of NMDAR channels to trigger Ca^2+^ influx (15, 72). In neuropathic pain, the increased synaptic expression of α2δ-1-NMDAR complex is essential for the enhancement of synaptic NMDAR activity, and the synaptic NMDAR hyperactivity can be reversed by interrupting the α2δ-1-NMDAR interaction (15, 16, 64). The importance of the α2δ-1 C-terminus in the induction of neuropathic pain has been shown using α2δ-1 chimera in which the C-terminus is mutated (73).

## 5. α2δ-1-NMDAR complex and ischemic stroke

### 5.1. The effects of gabapentinoids in ischemic stroke

Gabapentin was initially developed as an anticonvulsant, but it is also used to treat neuropathic pain. Gabapentin has been shown to reduce acute ischemic seizures, post-stroke pain, and spreading depression in brain injury (74–76). In an animal model of ischemic injury in the immature brain, gabapentin significantly decreases the severity of brain atrophy and acute seizures (74). The neuroprotective effects of gabapentinoids in ischemic stroke have been shown in various animal models (Supplementary Table 2). In a mouse model of transient focal ischemia, gabapentin pretreatment reduces the infarct volume by 23% independent of peri-infarct depolarization suppression (75). In patients with thalamic pain syndrome, gabapentin reduces the pain severity and the thalamus impairment (76). In the *in vitro* oxygen-glucose deprivation (OGD) model, gabapentin has a protective effect against neuronal injury (77). Systemic treatment of gabapentin reduces middle cerebral artery occlusion-induced infarct volumes, neurological deficit scores, and apoptosis (25). Furthermore, the neuroprotective effects of pregabalin on cerebral ischemia have been reported in a rodent stroke model, including the suppression of calcium-mediated proteolysis and the damage of oxidative stress, the attenuation of inflammation, and improving axon regeneration and motor outcome (78–81). Gabapentin and pregabalin have been extensively used in patients with chronic pain and anxiety disorders, exhibiting an excellent safety profile (82, 83). Thus, gabapentinoids could be repurposed for treating ischemic stroke in future.

### 5.2. The new insight of α2δ-1 in ischemic stroke

It has been reported that α2δ-1 may bind to thrombospondin, an astrocyte-secreted protein, to promote synaptogenesis (84). Gabapentin may reduce α2δ-1 interaction with thrombospondin and inhibit the new synapse formation (85). Pregabalin treatment induces axon sprouting and functional recovery in a mouse model of cortical stroke (81). However, astrocyte-derived thrombospondin-1 is upregulated in the astroglial peri-infarct scar but not elevated in remote cortical projection areas (81). This interaction may not account for the relatively rapid onset of gabapentinoid effects on acute cerebral ischemia. Another study suggested that the association between thrombospondin and α2δ-1 is rather weak, and no obvious α2δ-1-thrombospondin interaction can be detected on the cell surface (86).

During cerebral ischemia, excessive release of glutamate from presynaptic terminals can result in sustained Ca^2+^ influx through post-synaptic NMDARs and VGCCs. The neuroprotection by pregabalin was suggested to be associated with targeting VGCCs (78). The levels of α2δ-1 subunit can be detected in serum specimens, and the serum levels of α2δ-1 are significantly higher in ischemic stroke patients (87). Nevertheless, as mentioned above, gabapentinoids have no effect on VGCC activity *in vitro* and in neural tissues. Moreover, nimodipine, a widely used VGCC antagonist, has no efficacy in stroke patients as L-type Ca2^+^ channels are mainly distributed in cell bodies and proximal dendrites of neurons (88, 89).

**Figure 1:**
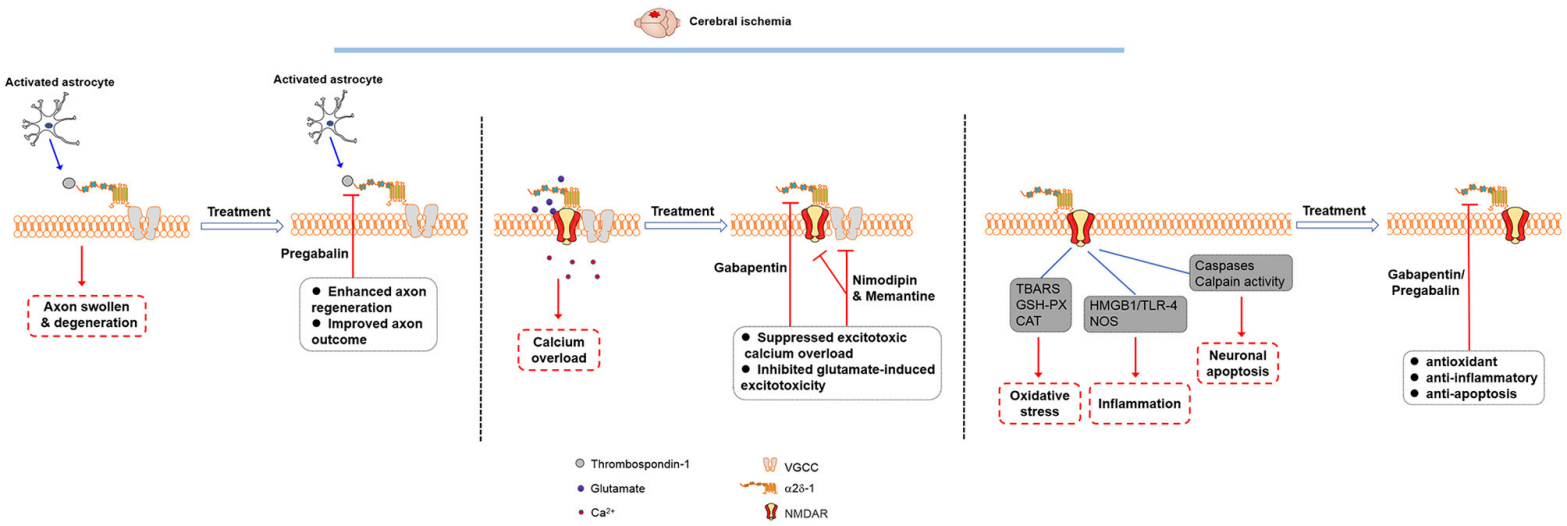
Molecular mechanisms involved in the therapeutic effects of gabapentinoids in cerebral ischemia. The thrombospondin-α2δ-1 interaction may play a role in synaptogenesis, but it is unlikely involved in the gabapentinoid effects on acute cerebral ischemia. Gabapentinoids likely act on α2δ-1-bound NMDARs to reduce excitotoxic Ca^2+^ overload and glutamate-induced excitotoxicity as well as produce antioxidant, anti-inflammatory, and anti-apoptosis effects in cerebral ischemia.

Inhibiting α2δ-1 with gabapentin has a profound inhibitory effect on oxygen-glucose deprivation-induced NMDAR hyperactivity in hippocampal CA1 neurons (25). In a heterologous expression system, gabapentin inhibits NMDAR activity only when α2δ-1 is coexpressed (15). Thus, the action of α2δ-1 *in vivo* is predominantly related to its association with NMDARs, which account for the protective actions of gabapentinoids in ischemic stroke (Figure 1).

### 5.3. α2δ-1-bound NMDAR as a potential therapeutic target in ischemic stroke

**Figure 2:**
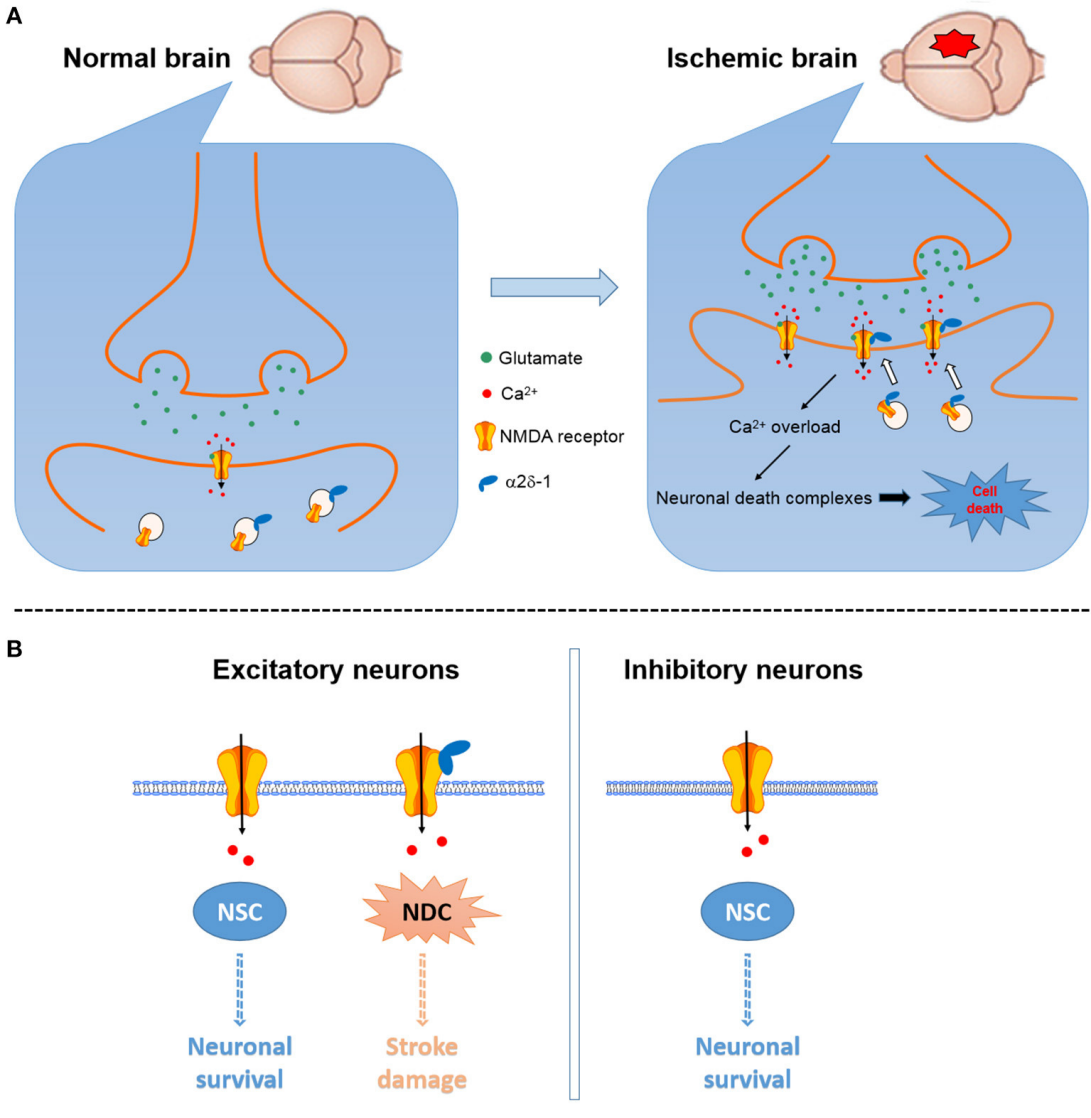
Schematic showing the potential role of α2δ-1 in NMDAR-mediated excitotoxicity in cerebral ischemia and reperfusion. **(A)** Cerebral ischemia can increase α2δ-1 expression and α2δ-1-NMDAR interactions to promote the synaptic trafficking of α2δ-1-bound NMDARs, resulting in NMDAR hyperactivity, calcium overload, and downstream cell death-signaling pathways and eventually neuronal death and brain damage [*modified from* Luo et al. (25)]. **(B)** α2δ-1-free NMDARs present in excitatory and inhibitory neurons likely mainly mediate the physiological functions of NMDARs via the neuronal survival-signaling complex (NSC), whereas α2δ-1-bound NMDARs expressed in excitatory neurons may predominantly activate the neuronal death-signaling complex (NDC).

α2δ-1 can readily form a heteromeric protein complex with phosphorylated NMDARs mainly through its C-terminus domain (15, 72). In the striatum, α2δ-1-bound NMDARs account for ~44% NMDARs present on the plasma membrane (90). In *Cacna2d1* knockout mice, transient cerebral ischemia does not increase the basal NMDAR currents, suggesting that the α2δ-1 may be essential for ischemia-induced neuronal NMDAR hyperactivity in the brain tissue (25). Accordingly, ischemia can increase the α2δ-1-NMDAR association, and α2δ-1-bound NMDARs mediate brain damage caused by cerebral ischemia or intracerebral hemorrhage (25, 69). Because α2δ-1 is preferentially expressed in excitatory neurons (47), α2δ-1-bound NMDARs may be the critical component for the NMDAR-mediated excitotoxicity (Figure 2). Targeting the α2δ-1-bound NMDARs using specific α2δ-1 C-terminus peptides or inhibitors would not interfere with the physiological, α2δ-1-free NMDAR function. Thus, α2δ-1-bound NMDARs could be targeted for the development of new neuroprotective drugs for treating and preventing ischemic stroke, including patients undergoing major neurological and cardiac surgeries.

## 6. Conclusion and implication

In summary, recent findings about α2δ-1-bound NMDARs have greatly advanced our understanding of the molecular mechanism of excitotoxicity associated with ischemic stroke. Compared with traditional non-selective NMDAR antagonists, treatment with α2δ-1 competing peptides or inhibitors (e.g., gabapentinoids) may represent an effective therapy for ischemic stroke. The subunit composition, synaptic localization, and numbers of NMDARs are not static but are dynamically regulated in response to neuronal activities (11). Further clinical research is needed to determine whether α2δ-1-bound NMDARs are a valid target for treating ischemic stroke.

## Author contributions

YL and H-LP contributed to the conception and design of the study. TW and YL wrote the first draft of the manuscript and performed the literature search. H-LP and S-RC critically revised the manuscript. All authors contributed to the article and approved the submitted version.

## Supplementary material

Supplementary Figure 1 Potential dual roles of NMDARs in neuronal survival and death. **(A)** NMDARs present at synaptic and extrasynaptic sites, which may be differentially involved in neuronal survival and death. **(B)** GluN2A- and GluN2B-containing NMDARs may have a different role in neuronal survival and death-signaling via the activation of downstream neuronal survival-signaling complex (NSC) and the activation of neuronal death-signaling complex (NDC).

Supplementary Table 1 The potential roles of α2δ-1-NMDAR complexes in various disease conditions.

Supplementary Table 2 Therapeutic effects of gabapentinoids in ischemic brain injury.
