# Supplementary figures and images for: The α2δ-1-NMDA receptor complex and its potential as a therapeutic target for ischemic stroke
Source: Front Neurol. 2023 Apr 20;14:1148697. doi: 10.3389/fneur.2023.1148697 (PMC10157046; doi:10.3389/fneur.2023.1148697)

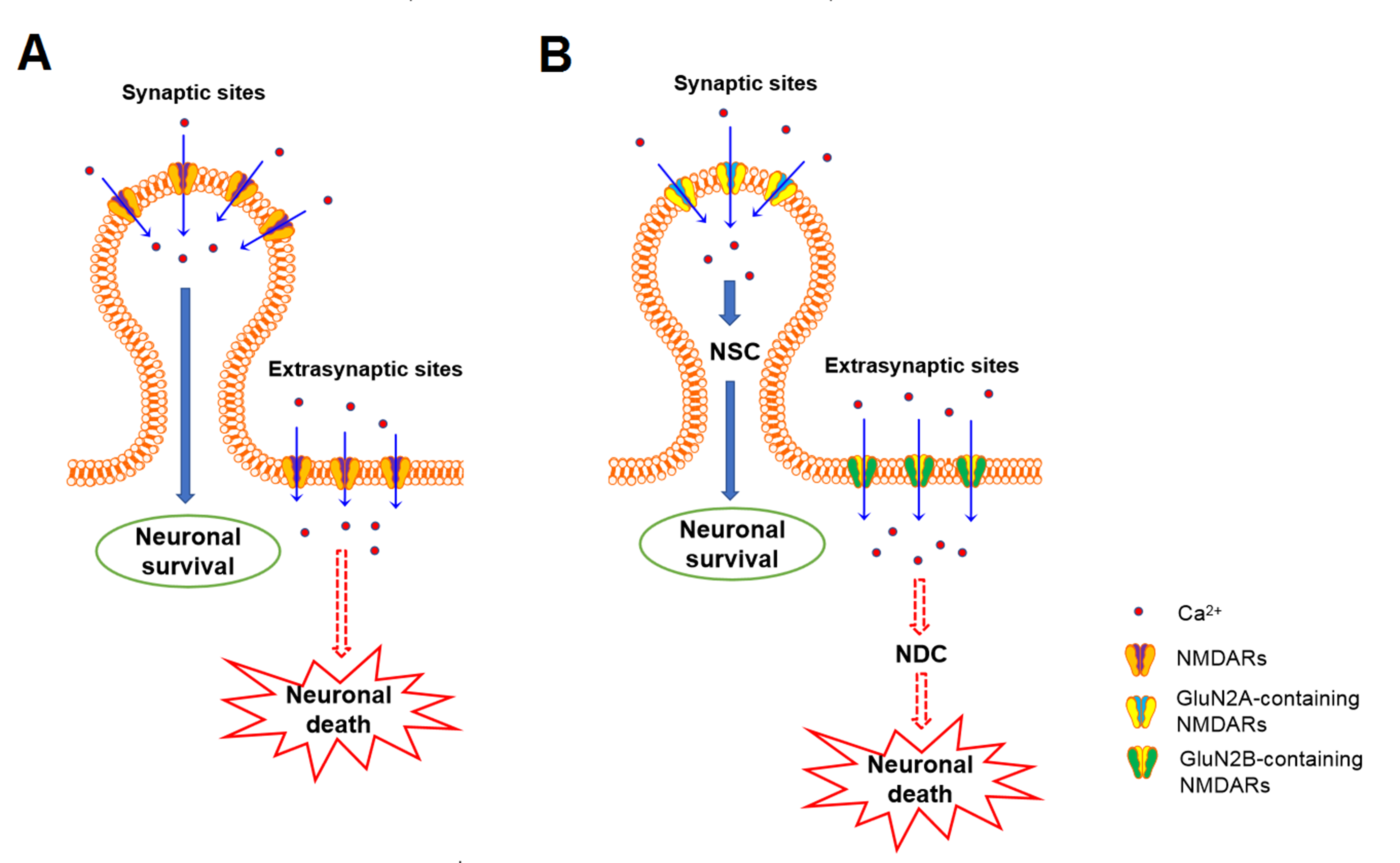

Supplement: Supplementary Figure 1 — Potential dual roles of NMDARs in neuronal survival and death. (A) NMDARs present at synaptic and extrasynaptic sites, which may be differentially involved in neuronal survival and death. (B) GluN2A- and GluN2B-containing NMDARs may have a different role in neuronal survival and death-signaling via the activation of downstream neuronal survival-signaling complex (NSC) and the activation of neuronal death-signaling complex (NDC). [file Image_1.TIF]
