# Supplementary material for: The α2δ-1-NMDA receptor complex and its potential as a therapeutic target for ischemic stroke
Source: Front Neurol. 2023 Apr 20;14:1148697. doi: 10.3389/fneur.2023.1148697 (PMC10157046; doi:10.3389/fneur.2023.1148697)
Supplement: Supplementary Table 1 — The potential roles of α2δ-1-NMDAR complexes in various disease conditions. [file Table_1.DOC]

**Table S1. The potential roles of α2δ-1-NMDAR complexes in various disease conditions**

| **Neuropathologies** | **Findings** | **Mechanisms** |
| --- | --- | --- |
| Neurogenic hypertension | SHRs are associated with increased α2δ-1 -NMDAR complexes in the hypothalamus in SHRs.  Chronic stress increases the expression of α2δ-1 and promotes physical interaction with and synaptic trafficking of NMDARs in the hypothalamus. | α2δ-1-bound NMDARs in the PVN are required for the potentiated presynaptic and postsynaptic NMDAR activity of PVN presympathetic neurons and for the elevated sympathetic outflow in hypertension ^[58]^.  α2δ-1-dependent NMDAR activity in the hypothalamus is an effector of genetic-environment interactions ^[59]^. |
| Ischemic stroke | Cerebral ischemia rapidly enhances the α2δ-1-NMDAR physical interaction in the brain in a mouse MCAO model. | α2δ-1 is essential for ischemia-induced neuronal NMDAR hyperactivity, and α2δ-1-bound NMDARs mediate brain injury caused by ischemia ^[60]^. |
| neuropathic pain | α2δ-1 is an NMDAR-interacting protein that increases NMDAR synaptic delivery in neuropathic pain.  Paclitaxel treatment potentiates the α2δ-1-NMDAR interaction and synaptic trafficking in the spinal cord.  TBS-driven synaptic plasticity required α2δ-1 and its interaction with NMDARs.  α2δ-1 upregulation after nerve injury is long lasting. | The α2δ-1-NMDAR interaction occurs through the C-terminus of α2δ-1 and promotes surface trafficking and synaptic targeting of NMDARs ^[15]^.  α2δ-1 is required for paclitaxel-induced tonic activation of presynaptic NMDARs at the spinal cord level ^[16]^.  TBS of sensory nerves induced persistent pain, which was maintained by α2δ-1-bound NMDARs ^[61]^.  Restoring the repressive HDAC2 function at the α2δ-1 gene promoter in primary sensory neurons leads to long-lasting relief of nerve pain ^[62]^. |
| Opioid-induced hyperalgesia & analgesic tolerance | Chronic morphine exposure increases the physical interaction between α2δ-1 and NMDARs. | α2δ-1-bound NMDARs contribute to opioid-induced LTP and hyperalgesia and tolerance by augmenting presynaptic NMDAR expression and activity at the spinal cord level ^[63, 64]^. |
| CIPS | Repeated treatment with tacrolimus (FK506) increases the amount of α2δ-1-NMDAR complexes in the spinal cord. | α2δ-1-bound NMDARs mediate calcineurin inhibitor-induced tonic activation of presynaptic and postsynaptic NMDARs at the spinal cord leve and play a major role in the development of CIPS ^[65]^. |
| Orofacial ectopic pain following nerve injury  ICH  PHN  Opioid misuse & addiction | Inferior alveolar nerve transection increases α2δ-1-NMDAR complex levels in the trigeminal ganglion in rats.  The expression levels of α2δ-1 and GluN1 were up-regulated in the mouse model of ICH.  RTX-induced neuropathy is associated with α2δ-1 upregulation in the DRG and increased α2δ-1-NMDAR physical interaction in the spinal cord.  Morphine treatment increases the physical interaction of α2δ-1 with NMDARs and their synaptic trafficking in the NAc. | The physical and functional interaction between α2δ-1 and NMDAR is critical for the development of orofacial ectopic pain ^[66]^.  The α2δ-1-NMDAR complex is involved in brain injury after ICH ^[67]^.  α2δ-1 promotes activation of presynaptic NMDARs and pain hypersensitivity in a nonviral animal model of PHN ^[68]^.  Repeated opioid exposure strengthens presynaptic and postsynaptic NMDAR activity in the NAc via α2δ-1 ^[69]^. |
| Abbreviations: NMDAR, *N*-methyl-_D_-aspartate receptor; SHR, spontaneously hypertensive rat; PVN, paraventricular nucleus; MCAO, middle cerebral artery occlusion; TBS, theta-burst stimulation; HDAC2, histone deacetylase-2; LTP, long-term potentiation; CIPS, calcineurin inhibitor-induced pain syndrome; ICH, intracerebral hemorrhage; PHN, postherpetic neuralgia; RTX, resiniferatoxin; DRG, dorsal root ganglion; NAc, nucleus accumbens. | | |
