# Supplementary material for: The α2δ-1-NMDA receptor complex and its potential as a therapeutic target for ischemic stroke
Source: Front Neurol. 2023 Apr 20;14:1148697. doi: 10.3389/fneur.2023.1148697 (PMC10157046; doi:10.3389/fneur.2023.1148697)
Supplement: Supplementary Table 2 — Therapeutic effects of gabapentinoids in ischemic brain injury. [file Table_2.DOC]

| **Gabapentinoids** | **Models/Subjects** | **Effects** |
| --- | --- | --- |
| Gabapentin | Right common carotid artery ligation surgery in postnatal day-12 mice | Gabapentin effectively reduces acute seizures and injury after ischemia in the immature brain ^[72]^. |
| Gabapentin | Mouse MCAO model | Gabapentin reduces infarct volume after ischemia independent of PID suppression ^[73]^. |
| Gabapentin | Patients with stroke in the thalamic area | Gabapentin may be considered as a first line therapy or as an add-on therapy for reducing the pain severity in patients with thalamic syndrome ^[74]^. |
| Gabapentin | OGD model in hippocampal CA1 neurons | Gabapentin has protective effects against glutamate-induced neuronal injury at least in part by inhibiting the NMDAR current ^[75]^. |
| Gabapentin | Mouse MCAO model | Systemic treatment with gabapentin reduced MCAO-induced infarct volumes, neurological deficit scores, and calpain/caspase-3 activation in brain tissues ^[60]^. |
| Pregabalin  Pregabalin  Pregabalin  Pregabalin | Mouse MCAO model  Rat model of carotid clamping and reperfusion  Rat model of hyperglycemic MCAO  Photothrombotic mouse model of cortical stroke | Pregabalin decreases the amount of Ca^2+^/calpain-mediated a-spectrin proteolysis in the cerebral cortex measured 6 hours post-MCAO ^[76]^.  Pregabalin protects the damage of oxidative stress after cerebral ischemia and reperfusion ^[77]^.  Pregabalin administration upon reperfusion decreases neuronal death and improved neurological function in hyperglycemic stroke rats, associated with attenuation of HMGB1/TLR-4-mediated inflammation and favorable modulation of the NOS ^[78]^.  Systemic pregabalin administration for 5 weeks after stroke augments novel peri-infarct motor cortex projections and improves skilled forelimb motor function ^[79]^. |
| Abbreviations: MCAO, middle cerebral artery occlusion; PID, peri-infarct depolarization; OGD, oxygen-glucose deprivation; NMDAR, *N*-methyl-_D_-aspartate receptor; HMGB1, high-mobility group box 1; TLR-4, toll-like receptor-4; NOS, nitric oxide synthase. | | |

**Table S2. Therapeutic effects of gabapentinoids in ischemic brain injury**
